# Characterization of a Metasurface Integrated 8-Plate Reconfigurable Coding Unit-Cell Coupler for Rotational Misalignment Resilience in UAV Wireless Power Transfer

**DOI:** 10.3390/mi17050620

**Published:** 2026-05-18

**Authors:** Jaewoo Jeong, Sangwook Park

**Affiliations:** 1 Department of ICT Convergence, Soonchunhyang Unversity, Asan 31538, Republic of Korea; andrea1019@sch.ac.kr; 2 Department of Electronic Engineering, Soonchunhyang Unversity, Asan 31538, Republic of Korea

**Keywords:** capacitive wireless power transfer, metasurface, digital coding, null power point, rotational misalignment, UAV wireless chrging

## Abstract

This study proposes a metasurface integrated reconfigurable unit-cell coupler designed for wireless power transfer (WPT) applications in unmanned aerial vehicles (UAVs). In near-field capacitive WPT systems, flexible UAV charging is restricted by rotational misalignment, which causes null power points (NPP) where energy transfer is suppressed. To address this, the proposed model emulates 1-bit digital coding states through Symmetric Excitation (SE) and Cross-Excitation (CE) states. Since precise unit-cell characterization is a prerequisite for array expansion, this research focuses on meta-atom-level analysis at 6.78 MHz with a deep sub-wavelength profile (0.002λ). Characterized through 3D full-wave analysis, the unit-cell achieves peak transmission coefficients of 0.945 for SE State and 0.903 for CE State. Crucially, these states exhibit complementary extinction angles at 90° and 45°, respectively, ensuring that the NPP of one state is effectively bypassed by the high transmissivity of the other. This dynamic switching between coding states maintains stable power transfer across a full 360° rotation, providing a technical foundation for scalable, intelligent metasurface-based wireless charging platforms.

## 1. Introduction

Metasurface technology presenting a new paradigm for electromagnetic wave control emerges as a key solution to revolutionize near-field wireless power transfer (WPT) systems [1,2]. The expanding industrial application of unmanned aerial vehicles (UAVs) necessitates wireless charging platforms providing stable energy regardless of position within the charging area. Rotational misalignment occurring during UAV landing significantly degrades coupling performance and remains a critical challenge [3,4]. Because UAVs approach from various directions, rotational displacement directly affects the alignment between interfaces to impact power transfer reliability [5]. So, integration of metasurface technology to actively reconfigure electromagnetic wavefronts is essential to ensure stable power transfer across all rotational states [6,7].

Integrating metasurfaces into near-field WPT enables spatial manipulation of electromagnetic fields to overcome physical constraints inherent in conventional electrode structures [8,9]. These ultra-thin structures implement artificial electromagnetic properties through periodic unit cell arrays to control boundary conditions [10]. Recent research expands the precise phase and amplitude manipulation of MHz-range near-fields beyond conventional far-field beamforming [11,12]. Reconfigurable metasurfaces utilizing digital coding techniques optimize electromagnetic responses in real time through dynamic unit cell state transitions [13,14]. Such active manipulation establishes a technological trajectory to compensate for performance degradation by reshaping energy coupling paths according to environmental dynamics.

Practical implementation of metasurface-based manipulation necessitates comprehensive analysis of physical constraints inherent in operational environments [15]. Maintaining constant transmission performance under diverse misalignment conditions is critical for system stability. Rotational misalignment between UAVs and charging interfaces alters the coupling area and induces a null power point (NPP) where mutual capacitance is effectively nullified [16,17]. This phenomenon requires system reliability and mandates resolution through active wavefront reconfiguration. Designing high-dimensional electromagnetic interfaces capable of controlling E-field symmetry is essential to ensure long-term robustness [18,19].

Implementing such interfaces requires projecting periodic unit cell arrays onto WPT structures. Inductive wireless power transfer (IPT) adds physical burden to periodic configurations due to the volume and weight of coils despite high power density [20]. Capacitive wireless power transfer (CPT) utilizing thin metal plates is more suitable for lightweight periodic arrays and system integration [21]. Conventional four-plate CPT structures however exhibit significant irregularity in coupling behavior under misalignment in parallel configurations and limited coupling characteristics in stacked configurations due to a low main-to-self-capacitance ratio [22,23]. New electrode structures functioning as meta-unit cells with maximized rotational resilience are therefore required to overcome these limitations.

Various studies attempted to modify coupler topologies to compensate for these structural limitations. Previous research introduced vertically aligned split circular couplers or concentric electrodes to enhance mutual capacitance stability under rotation and achieved an efficiency of 88.1% [24]. Furthermore, asymmetric designs for a vertically stacked four-plate structure controlled coupling coefficient variations within 5% while demonstrating stability at a 150 mm air gap [25]. However, these structural modifications remain limited to performance maintenance under specific conditions and suffer from implementation inefficiency due to structural complexity when expanded into periodic arrays through metasurface integration.

This paper proposes a metasurface integrated 8-plate CPT coupler overcoming irregular coupling characteristics through uniform deployment where plate connection states act as digital coding bits for dynamic wavefront reconfiguration. This active state transition shifts the NPP in space to avoid power interruption and ensures stable energy coupling across a full 360-degree rotational range; 3D full-wave analysis investigates transmission coefficient variations and equivalent capacitance networks to validate the proposed coupler. Vector analysis of near-field electromagnetic distributions and surface currents further confirms the wavefront control performance demonstrating coupling robustness achieved throughout the rotational range. These multifaceted analysis results establish a solid physical foundation for stable UAV wireless charging platforms utilizing the reconfigurable coding unit cell architecture.

## 2. Theoretical Analysis of the Proposed Metasurface Integrated 8-Plate CPT

### 2.1. Extension of the Conventional 4-Plate CPT to 8-Plate CPT

The proposed system utilizes a near-field metasurface interface where the 2 × 2 coplanar plate arrangement on each side serves as a single reconfigurable coding unit cell. While conventional four-plate CPT structures form a single displacement current loop, this unit-cell coupler establishes a dual-loop mechanism through two distinct coupling paths. The intra-unit-cell configuration dictates the loop directionality and subsequent power transfer characteristics. This serves as the primary mechanism for near-field wavefront manipulation.

**Figure 1:**
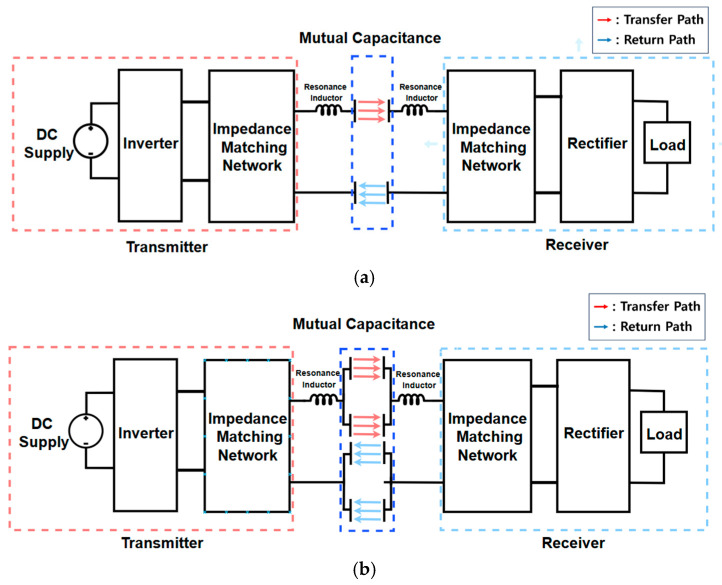

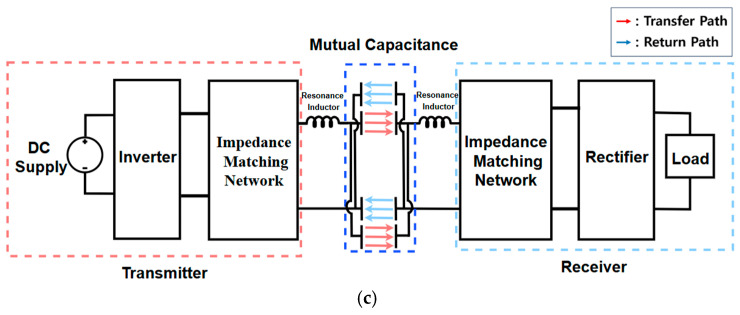
System Configuration of CPT: (**a**) 4-Plate CPT System; (**b**) 8-Plate CPT system with SE states; (**c**) 8-Plate CPT system with CE states.

Figure 1 shows the structural concept and overall configuration of the conventional four-plate CPT and the proposed metasurface-based CPT system. All configurations share a common power conversion topology. The transmitter side includes a DC source and a 6.78 MHz inverter to excite the transmitter unit cell. Resonant inductors are integrated into both sides to achieve the required resonance. In this study, the transfer coefficient exceeding 0.9 was achieved using only these resonant inductors. This high-Q coupling performance eliminates the need for impedance matching networks and validates the efficiency of the proposed coding unit-cell architecture.

As indicated in Figure 1, the combination of transfer and return paths defines the displacement current loops within the unit cell. The operational states of these loops depend on digital coding bits (0 or 1) assigned based on the electrical potential grouping of the sub-plate elements. This process enables active reconfiguration of the near-field response. The Symmetric Excitation (SE) coding state represents a constructive coding state where both loops share the same orientation. Conversely, the Cross-Excitation (CE) coding state acts as a differential coding state with counter-oriented loops. This coding-driven reconfiguration reconfigures the spatial electric field distribution to dynamically shift the null power point (NPP). Figure 1b,c show the operating cases for each coding state. This study demonstrates a reconfigurable coding strategy that exploits these complementary electromagnetic profiles to ensure rotational resilience across the 360° range.

### 2.2. Definition of SE and CE States in the Metasurface Integrated 8-Plate CPT

**Figure 2:**
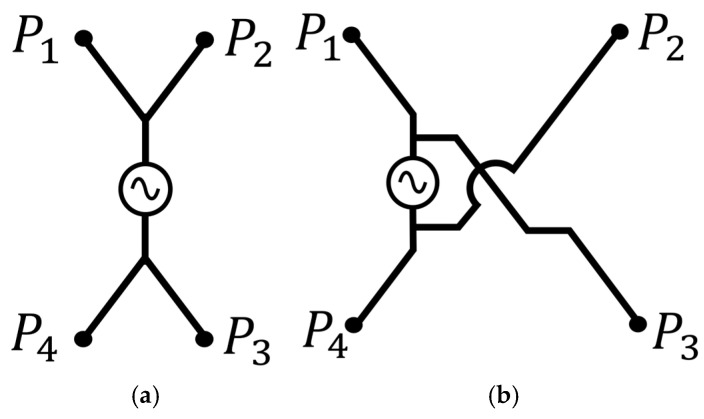
Schematic diagrams of the proposed switchable coding sequence for the metasurface integrated 8-plate CPT coupler: (**a**) SE states; (**b**) CE states.

This section defines the SE and CE states as discrete digital coding states within the 2 × 2 unit-cell architecture. Each excitation state is distinguished by the potential grouping of sub-plate elements determined by the specific coding sequence. Figure 2a shows the SE state. Plates $P_{1}$ and $P_{2}$ are assigned an identical coding bit in this state to form a single equipotential group while $P_{3}$ and $P_{4}$ constitute the opposite group. This parallel assignment establishes a constructive feeding structure where the potential is shared between the upper and lower plate pairs.

Figure 2b shows the CE state. This state groups plates along the diagonal direction into the same coding bit to form a cross-connected excitation structure. Plates $P_{1}$ and $P_{3}$ are paired specifically against $P_{2}$ and $P_{4}$ to create two separate equipotential groups. These unit-cell assignments fundamentally redistribute the potential across the entire metasurface interface to modulate the power transfer characteristics.

### 2.3. Node-Merging-Based Unified Modeling of the Equivalent Capacitance Network

**Figure 3:**
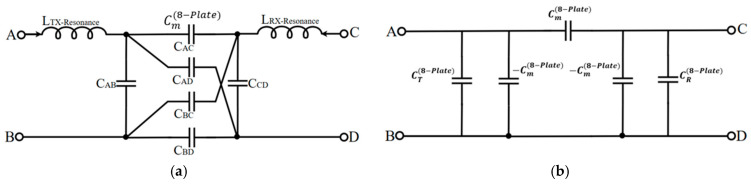
Equivalent circuit of the proposed metasurface integrated 8-plate CPT under node merging. (**a**) Equivalent circuit; (**b**) Pi Model.

Figure 3a shows the equivalent circuit of the metasurface integrated coupler as a two-port network and Figure 3b presents the corresponding capacitance Pi Model. This model utilizes node merging to merge the 2 × 2 coding unit cell into a simplified representation for NPP analysis. Distinguishing between coding states within the same equivalent circuit requires merging sub-plate elements with identical potentials into a single node. This unified framework simplifies the analysis of the capacitive network transitions occurring under rotational misalignment.

Each unit cell operates as a one-port structure comprising two potential groups. On the transmitter side, plates carrying positive and negative potentials define Node A and Node B, respectively. Node C and Node D are defined on the receiver side following the same convention. Transitions in the digital coding state alter the plate combinations for each node to redefine the equivalent capacitance network. Consequently, both coding states can be analyzed within a single unified equivalent circuit framework.

In the proposed equivalent circuit, the self-capacitance $C_{AB}$ and $C_{CD}$, cross-capacitance $C_{AD}$ and $C_{BC}$, and main capacitance $C_{AC}$ and $C_{BD}$ are each defined based on the node combination described above. Based on the four-node capacitance network defined above the equivalent self-capacitances $C_{T}^{\left(8\text{-}Plate\right)}$ and $C_{R}^{\left(8\text{-}Plate\right)}$ are expressed as follows:

(1) $$C_{T}^{\left(8\text{-}Plate\right)}=C_{AB}+\frac{{(C}_{AC}+C_{AD})(C_{BC}+C_{BD})}{C_{AC}+C_{AD}+C_{BC}+C_{BD}}$$

(2) $$C_{R}^{\left(8\text{-}Plate\right)}=C_{CD}+\frac{{(C}_{AC}+C_{BC})(C_{AD}+C_{BD})}{C_{AC}+C_{AD}+C_{BC}+C_{BD}}$$

The equivalent mutual capacitance $C_{m}^{\left(8\text{-}Plate\right)}$, which determines the power transfer efficiency between the transmitter and receiver sides, is defined as shown in Equation (3). This parameter is critical for identifying the origin of NPP. The rotational angle corresponding to the NPP shifts according to the assigned bit sequence since the sub-plate combinations constituting $C_{m}^{\left(8\text{-}Plate\right)}$ vary with the digital coding state.

(3) $$C_{m}^{\left(8\text{-}Plate\right)}=\frac{C_{AC}C_{BD}-C_{AD}C_{BC}}{C_{AC}+C_{AD}+C_{BC}+C_{BD}}$$

The resonant frequency for each port is defined as follows:

(4) $$f_{0,\;T}^{\left(8\text{-}Plate\right)}=\frac{1}{2\pi \sqrt{L_{TX\text{-}Resonance}C_{T}^{\left(8\text{-}Plate\right)}}}$$

(5) $$f_{0,\;R}^{\left(8\text{-}Plate\right)}=\frac{1}{2\pi \sqrt{L_{RX\text{-}Resonance}C_{R}^{\left(8\text{-}Plate\right)}}}$$

The selection of the digital coding sequence determines the node grouping and the configuration of cross-coupling components to alter the formation of $C_{m}^{\left(8\text{-}Plate\right)}$ as a function of rotational angle. Specifically, when the coupler reaches a rotational angle satisfying $C_{AC}C_{BD}=C_{AD}C_{BC}$, $C_{m}^{\left(8\text{-}Plate\right)}$ is made zero. This phenomenon represents the NPP condition where the electrical coupling between the transmitter and receiver unit cells vanished.

### 2.4. The 3D Simulation Model and Rotation Scenarios of the Proposed Metasurface Integrated 8-Plate CPT

**Figure 4:**
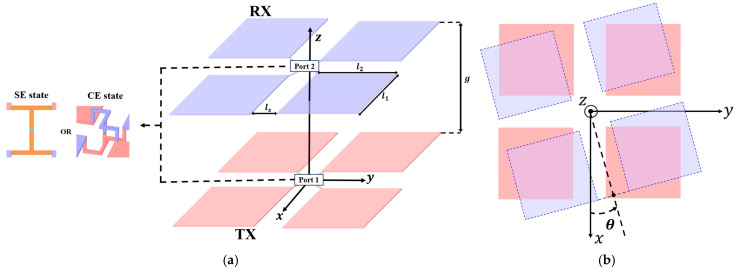
Simulation model and rotation scenario of the proposed metasurface integrated 8-plate CPT: (**a**) simulation model; (**b**) rotation scenario.

A 3D full-wave analysis using ANSYS HFSS 2021 R2 evaluates the coupling characteristics of the proposed coupler. Figure 4a shows the simulation model incorporating identical unit cell configurations on both transmitter and receiver interfaces where each sub-plate element consists of a copper sheet with uniform dimensions. Table 1 and Table 2 summarize the geometric parameters and the resonant inductor values required for achieving 6.78 MHz resonance, respectively. Previous studies demonstrate that 3D full-wave simulations exhibit high correlation with experimental measurements [26]. This research consequently validates system feasibility by analyzing transmission coefficient trends and electromagnetic field distributions across various rotational angles.

Coding sequence states were implemented by modifying only the electrical potential assignment of the sub-plates while maintaining a constant geometric structure. Each coding sequence was configured as an independent analysis model to verify the reconfigurable response of the unit cell under various rotational scenarios.

Figure 4b presents a top view of the analysis model to illustrate the rotational misalignment scenario. This study assumes the receiver unit cell rotates in the XY plane with the Z-axis as the axis of rotation. The rotational angle θ defines the relative angular displacement of the receiver sub-plates with respect to the transmitter interface. The rotation follows a positive direction to evaluate the spatial resilience of the reconfigurable coding unit cell.

For convenience of case classification, the representative rotational angles of 0°, 45° and 90° for each mode are defined as Case SE-0, Case SE-45 and Case SE-90 for SE state and Case CE-0, Case CE-45 and Case CE-90 for CE state, respectively. Operating scenarios for each representative angle were established to investigate the coupling performance under rotational misalignment as summarized in Table 3.

The SE state is expected to maintain stable coupling in Case SE-0 and Case SE-45, whereas NPP is predicted to occur in Case SE-90. In contrast, the CE state exhibits stable coupling in Case CE-0 and Case CE-90 while NPP is anticipated in Case CE-45. To validate these predictions, 3D full-wave analysis was conducted to investigate the correlation between the transmission coefficient and the equivalent mutual capacitance. These analyses further visualized the electromagnetic field and surface current vector distributions to elucidate the physical mechanism of the reconfigurable unit cell.

## 3. Results and Discussion

### 3.1. The 3D Full-Wave Simulation Results

#### 3.1.1. Comparison of Transmission Coefficient Responses at Representative Rotation Angles in SE State and CE State

**Figure 5:**
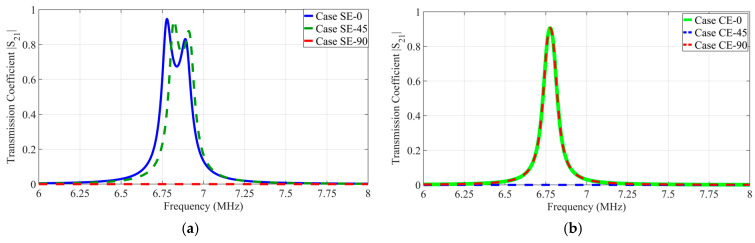
Comparison of transmission coefficient at representative rotation angles $\left(0^{{}^{\circ}},45^{{}^{\circ}},90^{{}^{\circ}}\right)$ for the proposed 8-plate CPT system: (**a**) SE state; (**b**) CE state.

Figure 5a shows the transmission coefficient characteristics of the SE state as a function of the representative rotational angle. Simulation results confirm that Case SE-0 and Case SE-45 maintain high transmission coefficients of 0.9453 at 6.78 MHz and 0.9377 at 6.82 MHz, respectively. These coupling characteristics arise from the formation of a constructive coding state where the coupling components of the two channels superimpose with identical polarity. This ensures sufficient equivalent mutual capacitance between the unit cells.

This process induces a frequency splitting phenomenon where the single resonant point separates into two distinct resonant modes. The system maintains high coupling performance at each split resonant frequency due to the strong interaction between the metasurface interfaces. In contrast, Case SE-90 exhibits a transmission coefficient converging to zero at 6.78 MHz. This result indicates that a 90° rotation in the SE state satisfies the NPP condition where the mutual capacitance is nullified. The coupling performance is expected to recover beyond this angular displacement as the unit-cell symmetry shifts.

Figure 5b shows transmission coefficient characteristics of the CE state as a function of representative rotational angles. Simulation results confirm that Case CE-0 and Case CE-90 maintain a peak transmission coefficient of 0.9028 at 6.78 MHz. Conversely, Case CE-45 converges to zero at 6.78 MHz, indicating that a 45° rotation in CE state satisfies the NPP condition. These results demonstrate that the proposed 8-plate CPT coupler possesses complementary NPP distributions according to coding states to effectively eliminate power transmission dead zones throughout the rotational range.

#### 3.1.2. Correlation Between Rotational Transmission Coefficient and Mutual Capacitance in SE State and CE State

A parametric sweep analysis investigated the operational continuity and robustness of the proposed coupler across the full rotational range from 0° to 180°. This process analyzed the variation in the maximum transmission coefficient and equivalent mutual capacitance to validate the spatial resilience of the coding unit cell.

**Figure 6:**
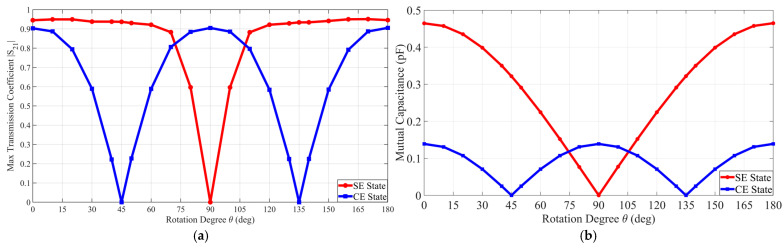
Comparison of the maximum transmission coefficient and mutual capacitance according to the rotational misalignment angle in the metasurface integrated 8-plate CPT under SE state and CE state conditions: (**a**) maximum transmission coefficient; (**b**) mutual capacitance.

Figure 6a displays the maximum transmission coefficient as a function of the rotational angle for each coding state. The SE state maintains a high transmission coefficient in the ranges of 0° to 70° and 110° to 180°  but nullifies at 90°. Conversely the CE state exhibits a reduction to zero at 45° and 135° while recovering peak coupling performance at 90°.

Figure 6b compares the mutual capacitance variation for each digital coding state. Consistent with the transmission coefficient trends the mutual capacitance for the SE state converges to 0 pF at 90°. The CE state similarly reaches a null point at 45° and 135°. Table 4 and Table 5 summarize the specific capacitance values extracted for each coding state, respectively.

### 3.2. Physical Mechanism Analysis via Electromagnetic Wavefront Reconfiguration

#### 3.2.1. Electric Vector Distribution Analysis of the Proposed Metasurface Integrated 8-Plate CPT Under SE State

This section performs a 3D full-wave analysis based on electromagnetic physics to clarify the operating principle of the proposed coupler and the coupling suppression phenomenon caused by rotational misalignment. To establish a comparative basis, the aligned condition and the NPP condition are examined in contrast. Specifically, Case SE-0 and Case SE-90 represent the SE state while Case CE-0 and Case CE-45 compare the CE state.

This analysis examines surface current vectors based on current density and directionality along the internal paths of the unit-cell interface. Electric field vectors are analyzed to make clear the mutual coupling mechanism and field formation between the transmitter and receiver unit cells. This vector-based visualization identifies the physical causal relationship where the electric field coupling is nullified at specific rotational angles. This electromagnetic perspective clarifies the mechanism of energy coupling suppression resulting from wavefront mismatch.

An observation sheet crossing a specific cross-section was established to precisely visualize the magnitude and directionality of the electric field vectors formed between the unit cells. The sheet dimensions were 200 mm in width and 140 mm in height positioned on the XY plane offset by 27.5 mm in the negative y-direction. This area ensures complete observation of the electric field distribution including fringing effects at the sub-plate edges to provide a comprehensive analysis of the wavefront reconfiguration.

**Figure 7:**
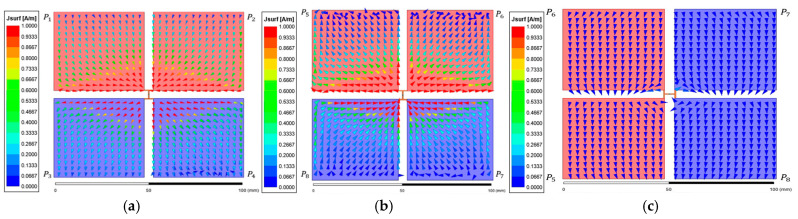
Surface current vector distribution of the metasurface integrated 8-plate CPT under SE state: (**a**) top view of the transmitter; (**b**) top view of the receiver in Case SE-0; (**c**) top view of the receiver in Case SE-90.

Figure 7 shows the surface current vector distribution under the SE state. Figure 7a presents the transmitter interface while Figure 7b,c illustrate the receiver side distributions for Case SE-0 and Case SE-90, respectively. On the transmitter side, two internal current paths formed between the sub-plate pairs ($P_{1}$, $P_{2}$ and $P_{3}$, $P_{4}$) share an identical potential and are directed downward. Case SE-0 demonstrates strong electrostatic coupling between the unit-cell interfaces under the aligned condition. Induced currents are formed within the receiver sub-plates in the opposite direction to the transmitter paths following the electric field coupling principle. This symmetric current distribution indicates that the displacement current flow is optimized through effective wavefront alignment. In contrast, Case SE-90 exhibits a significantly reduced current density within the receiver sub-plates because the structural mismatch caused by 90° rotation leads to the nullification of the coupling.

**Figure 8:**
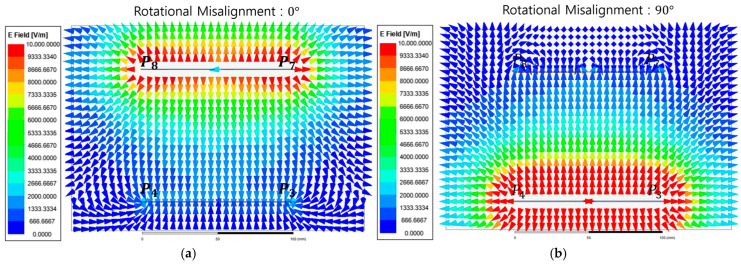
Instantaneous electric field vector distributions of the metasurface integrated 8-plate CPT in the SE state at Phase 0°: (**a**) Case SE-0; (**b**) Case SE-90.

Figure 8 shows the electric field vector distribution between the transmitter and receiver unit-cell interfaces at Phase 0°. Figure 8a shows the aligned condition where electric field vectors originating from transmitter sub-plates $P_{3}$ and $P_{4}$ are strongly concentrated in the vertical direction toward receiver sub-plates $P_{7}$ and $P_{8}$. This formation of a high-density coupling path physically demonstrates an effective potential difference, enabling optimal energy coupling. Conversely Figure 8b shows the NPP condition. Despite the presence of a strong electric field near the transmitter, the electric field vector density near the receiver sub-plates decreases sharply and is effectively nullified.

#### 3.2.2. Electric Vector Distribution Analysis of the Proposed Metasurface Integrated 8-Plate CPT Under CE State

**Figure 9:**
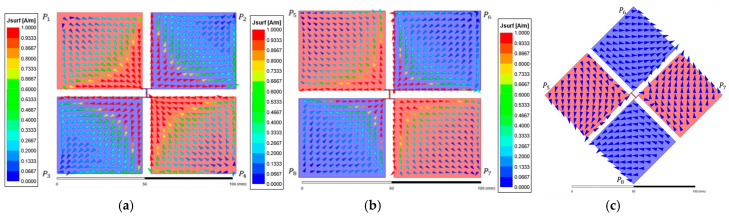
Surface current vector distribution of the metasurface integrated 8-plate CPT under CE state: (**a**) top view of the transmitter; (**b**) top view of the receiver in Case CE-0; (**c**) top view of the receiver in Case CE-45.

Figure 9 presents the surface current vector distribution analysis under the CE state. Figure 9a shows the reference vector distribution of the transmitter interface while Figure 9b and Figure 9c show the receiver side distributions for Case CE-0 and Case CE-45, respectively. On the transmitter interface, sub-plate elements forming diagonal equipotential pairs ($P_{1}$, $P_{3}$ and $P_{2}$, $P_{4}$) exhibit opposing downward and upward internal current paths. This is consistent with the differential coding state of the unit cell where opposing current directions generate a differential electric field profile. Case CE-0 exhibits high-density induced currents within the receiver sub-plates in the opposite direction to the transmitter paths. In contrast, Case CE-45 under the NPP condition shows that the differential electric field is nullified at the receiver interface due to the breakdown of structural symmetry. The formation of effective induced currents is consequently suppressed across the receiver sub-plates, resulting in a significantly reduced electric field distribution. This provides primary physical evidence that a 45° rotation in the CE state corresponds to the NPP condition where electric coupling is physically inhibited.

**Figure 10:**
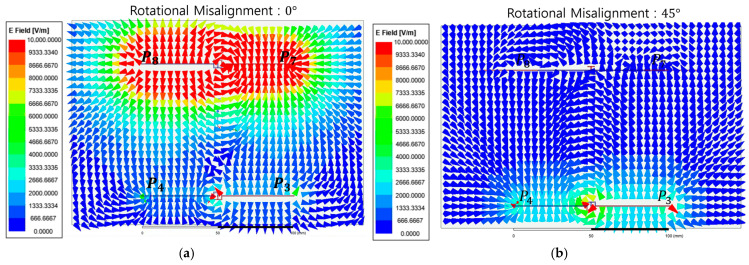
Instantaneous electric field vector distributions of the metasurface integrated 8-plate CPT in the CE state at Phase 0°. (**a**) Case CE-0; (**b**) Case CE-45.

Figure 10 shows the electric field vector distribution between the unit-cell interfaces under the CE state at Phase 0°. Figure 10a shows the aligned condition where the electric field at sub-plate P8 is directed outward while the field at the adjacent sub-plate P7 is directed inward. This specific field directionality demonstrates that the differential electric field flux generated by the transmitter unit cell is strongly coupled with the receiver interface to form an effective coupling loop. In contrast, Figure 10b shows the NPP condition. A strong electric field remains visible near the transmitter interface yet the electric field vector density near the receiver sub-plates decreases sharply. This indicates that the energy coupling to the receiver unit cell is effectively nullified due to the phase-canceling characteristics of the differential coding state under rotational mismatch.

## 4. Conclusions

This research proposes a metasurface integrated 8-plate digital coding unit cell coupler to fundamentally resolve rotational misalignment and resulting NPP issues in UAV wireless charging platform. Unified circuit modeling based on node merging was performed to clarify the physical operation of the proposed meta-atom and 3D full-wave analysis precisely investigated the rotation dependency of the transmission coefficient and mutual capacitance network at 6.78 MHz. Analysis results confirmed complementary electromagnetic responses where the Symmetric Excitation coding state maintains a peak transmission coefficient of 0.9453 at 0-degree alignment but nullifies at 90 degrees while the Cross-Excitation coding state reaches peak coupling at 90 degrees and induces NPP at 45 degrees. Dynamic transition between these 1-bit digital coding states actively shifts the NPP in space to avoid energy interruption zones and provides a technical path ensuring robust coupling across a full 360-degree rotational range. The unit cell characterization results obtained in this study will serve as essential meta-atom analysis metrics for future expansion into large-scale intelligent metasurface-based charging platforms. Future research will focus on fabricating a hardware prototype integrated with power conversion circuits to measure system efficiency and conducting studies on charging area expansion and wavefront control optimization through unit cell array configurations.

## Figures and Tables

**Table 1:** Physical and geometric parameters of the simulation model for the metasurface integrated 8-plate CPT.

Components	Notation	Value
Copper Plate Width	$l_{1}$	50 mm
Copper Plate Length	$l_{2}$	50 mm
Spacing Between Copper Plate	$l_{s}$	5 mm
Distance Between TX and RX	g	90 mm

**Table 2:** Resonant inductor parameter values for the metasurface integrated 8-plate CPT system.

Parameter	Value
SE State Lumped Inductor	153.92 μH
CE State Lumped Inductor	112.92 μH

**Table 3:** Predictive evaluation of coupling performance under rotational misalignments.

	Degree	0°	45°	90°
Case				
Case SE		○	○	×
Case CE		○	×	○

**Table 4:** Plate capacitance values under rotational misalignment in the SE state.

	0°	45°	90°	135°	180°
$C_{AC}$	0.54 pF	0.50 pF	0.42 pF	0.34 pF	0.30 pF
$C_{BD}$	0.54 pF	0.50 pF	0.42 pF	0.34 pF	0.31 pF
$C_{AD}$	0.30 pF	0.34 pF	0.42 pF	0.50 pF	0.54 pF
$C_{BC}$	0.30 pF	0.34 pF	0.42 pF	0.50 pF	0.54 pF
${(C}_{AC}C_{BD})-(C_{AD}C_{BC})$	0.19 pF	0.13 pF	0 pF	0.13 pF	0.19 pF
$C_{m}$	0.11 pF	0.08 pF	0 pF	0.08 pF	0.11 pF

**Table 5:** Plate capacitance values under rotational misalignment in the CE state.

	0°	45°	90°	135°	180°
$C_{AC}$	0.46 pF	0.42 pF	0.39 pF	0.42 pF	0.46 pF
$C_{BD}$	0.46 pF	0.42 pF	0.39 pF	0.42 pF	0.46 pF
$C_{AD}$	0.39 pF	0.42 pF	0.46 pF	0.42 pF	0.39 pF
$C_{BC}$	0.39 pF	0.42 pF	0.46 pF	0.42 pF	0.39 pF
${(C}_{AC}C_{BD})-(C_{AD}C_{BC})$	0.05 pF	0 pF	0.05 pF	0 pF	0.05 pF
$C_{m}$	0.03 pF	0 pF	0.03 pF	0 pF	0.03 pF

## Data Availability

The raw data supporting the conclusions of this article will be made available by the authors upon request.

## References

[1] Lee W., Yoon Y.-K. (2022). High Efficiency Multiscale Wireless Power Transfer System Using Metasurface Slabs. IEEE Access.

[2] Rong C., Yan L., Li L., Li Y., Liu M. (2023). A Review of Metamaterials in Wireless Power Transfer. Materials.

[3] Aydin E. (2025). A Misalignment Tolerant Magnetic Coupler Design and Implementation for Wireless Charging of Unmanned Aerial Vehicles (UAVs). IEEE Access.

[4] Chang J., Cai W., Wang H., Guo Y., Wu J., Rong C., Xia C. (2025). A Wireless Power Transfer System for Unmanned Aerial Vehicles with CC/CV Charging Based on Topology Switching. Appl. Sci..

[5] Campi T., Cruciani S., Maradei F., Feliziani M. (2021). Efficient Wireless Drone Charging Pad for Any Landing Position and Orientation. Energies.

[6] Li K., Naderi M.Y., Muncuk U., Chowdhury K.R. (2023). MetaResonance—A Reconfigurable Surface for Holographic Wireless Power Transfer. IEEE Trans. Ind. Electron..

[7] Zhang Z., Shi H., Wang L., Chen J., Chen X., Yi J., Zhang A., Liu H. (2023). Recent Advances in Reconfigurable Metasurfaces: Principle and Applications. Nanomaterials.

[8] Markvart A., Song M., Glybovski S., Belov P., Simovski C., Kapitanova P. (2020). Metasurface for Near-Field Wireless Power Transfer With Reduced Electric Field Leakage. IEEE Access.

[9] Hiep L.T.H., Khuyen B.X., Tung B.S., Ngo Q.M., Lam V.D., Pham T.S. (2022). Flexible Magnetic Metasurface with Defect Cavity for Wireless Power Transfer System. Materials.

[10] Barbarić D., Šipuš Z. (2020). Designing Metasurfaces with Canonical Unit Cells. Crystals.

[11] Lee W., Yoon Y.-K. (2023). High-Efficiency Wireless-Power-Transfer System Using Fully Rollable Tx/Rx Coils and Metasurface Screen. Sensors.

[12] Huu Nguyen B., Thanh Son P., Hiep L.T.H., Anh N.H., Tung D.K., Khuyen B.X., Tung B.S., Lam V.D., Zheng H., Chen L. (2024). Enhancing Wireless Power Transfer Performance Based on a Digital Honeycomb Metamaterial Structure for Multiple Charging Locations. Crystals.

[13] Gao L., Zhou Y., Zhu H., Zheng P., Liu J., He Z., Xu Z., Cui Y. (2023). Reconfigurable Amplitude-Phase-Coding Metasurface with Flexible Beamforming Capability. Electronics.

[14] Fazal D., Hong I.-P. (2023). A New Unit-Cell Design for a 2-Bit Reflective Metasurface for RIS Applications. Electronics.

[15] Wang S., Jiang C., Tao X., Chen F., Rong C., Lu C., Zeng Y., Liu X., Liu R., Wei B. (2020). Enhancing the Stability of Medium Range and Misalignment Wireless Power Transfer System by Negative Magnetic Metamaterials. Materials.

[16] Bang K., Bae H., Park S. (2023). Resonant-Based Wireless Power Transfer System Using Electric Coupling for Transparent Wearable Devices and Null Power Points. Sensors.

[17] Bae H., Park S. (2025). Design of Four-Plate Parallel Dynamic Capacitive Wireless Power Transfer Coupler for Mobile Robot Wireless-Charging Applications. Appl. Sci..

[18] Rouse C.D., Cove S.R., Salami Y., Arsenault P., Bartlett A. (2022). Three-Phase Resonant Capacitive Power Transfer for Rotary Applications. IEEE J. Emerg. Sel. Top. Power Electron..

[19] Bae H.-G., Park S.-W. (2025). Design and Theoretical Analysis of a Hexagonal-Stacked MISO Electric Resonant Coupling Wireless Power Transfer Coupler. Electronics.

[20] Van Ieperen A., Derammelaere S., Minnaert B. (2026). Empirical Performance Survey of Inductive and Capacitive Wireless Power Transfer Systems. Electronics.

[21] Wang Z., Zhang Y., He X., Luo B., Mai R. (2022). Research and Application of Capacitive Power Transfer System: A Review. Electronics.

[22] Lecluyse C., Minnaert B., Kleemann M. (2021). A Review of the Current State of Technology of Capacitive Wireless Power Transfer. Energies.

[23] Bezawada Y., Dhali S.K. (2022). 8-Plate Multi-Resonant Coupling Using a Class-E2 Power Converter for Misalignments in Capacitive Wireless Power Transfer. Electronics.

[24] Park C., Park J., Shin Y., Kim J., Huh S., Kim D., Park S., Ahn S. (2020). Separated Circular Capacitive Coupler for Reducing Cross-Coupling Capacitance in Drone Wireless Power Transfer System. IEEE Trans. Microw. Theory Tech..

[25] Zhang H., Lu F., Hofmann H., Liu W., Mi C. (2016). A 4-Plate Compact Capacitive Coupler Design and LCL-Compensated Topology for Capacitive Power Transfer in Electric Vehicle Charging Applications. IEEE Trans. Power Electron..

[26] Kim J., Park S. (2025). Design of an Orthogonally Stacked DD Coil-Split Capacitive Plate Hybrid Coupler for UAV Wireless Charging. Appl. Sci..

